# Mutant Characterisation Reveals Two Candidate Sensors for the Rapid High Vapour Pressure Deficit (VPD) Response Pathway

**DOI:** 10.1111/pce.70857

**Published:** 2026-08-31

**Authors:** Hanh M. Vo, Timothy J. Brodribb, Frances C. Sussmilch

**Affiliations:** ^1^ School of Natural Sciences University of Tasmania Sandy Bay, TAS Australia

## Abstract

We investigated the rapid high VPD response pathway by characterising *Arabidopsis thaliana* mutants for five candidate genes: *ATX2, HVR, ILK1, MSL2*, and *OSCA1.2*, and identified *HVR* and *MSL2* as critical sensors mediating this rapid response.

Climate change has increased the frequency and severity of dehydration stresses, including high vapour pressure deficit (VPD), a rapidly fluctuating driver of transpiration through open stomata. Many angiosperms respond rapidly by synthesising the stress hormone abscisic acid (ABA), which induces stomatal closure and limits transpiration (e.g. McAdam et al. 2016).

Within minutes of high VPD exposure, transcription of genes encoding the rate‐limiting ABA biosynthesis enzyme nine‐*cis*‐epoxycarotenoid dioxygenase (NCED) (Qin and Zeevaart 1999) is rapidly triggered (McAdam et al. 2016). However, the genetic components linking high VPD to *NCED* expression remain uncharacterised. Most studies have focused on dehydration stress pathways over extended periods (hours – days), rather than the short signalling time frames associated with stomatal closure within 20 min of high VPD exposure (McAdam et al. 2016).

The pathway linking high VPD to ABA biosynthesis likely involves mechano‐ or osmo‐sensitive receptors that detect changes in cell turgor (Kalladan et al. 2017) and/or organelle osmotic potential (Wilson et al. 2014). The sensor(s) may activate intermediate signalling proteins that trigger *NCED* upregulation. A previous investigation excluded ARABIDOPSIS HISTIDINE KINASE1 as a major regulator of rapid *AtNCED3* induction in Arabidopsis (Sussmilch et al. 2017). The core components of this pathway remain uncharacterised.

We selected five candidates for the rapid high VPD response pathway in Arabidopsis. These are (i) AT1G34300, which encodes a β‐lectin receptor‐like kinase (RLK) protein (Kalladan et al. 2017); (ii) *INTEGRIN‐LINKED KINASE1* (*ILK1*), encoding a cell membrane‐localised protein (Brauer et al. 2016); (iii) *MECHANOSENSITIVE CHANNEL of SMALL CONDUCTANCE‐LIKE2* (*MSL2*), encoding a plastid membrane‐localised protein (Wilson et al. 2014); (iv) a cell membrane ion channel‐encoding gene *REDUCED HYPEROSMOLALITY‐INDUCED Ca* ^*2+*^ *INCREASE 1.2* (*OSCA1.2*; Hou et al. 2014); and v) *ARABIDOPSIS TRITHORAX‐LIKE2* (*ATX2*), encoding a histone methyltransferase (Liu et al. 2018).

The candidate sensors are membrane‐localised proteins. Previously, SNPs at the AT1G34300 locus were found to be associated with ABA accumulation, and loss‐of‐function mutants exhibited reduced water loss in detached leaves and higher ABA levels under osmotic stress compared to wild‐type (WT) plants (Kalladan et al. 2017). The cell plasma membrane‐localised kinase ILK1 plays a key role in hyper‐osmotic response (Brauer et al. 2016). MSL2 is essential for regulating plastid osmotic potential (Wilson et al. 2014). The *msl2* mutant exhibits higher *AtNCED3* levels than WT under standard conditions, indicating a role in suppressing *AtNCED3* expression under normal conditions (Wilson et al. 2014). *OSCA1.2* belongs to the hyperosmolality‐gated calcium‐permeable cation channel‐encoding *OSCA* family. OSCA1.2 is activated under hyper‐osmotic stress, mediating calcium ion influx, suggesting *OSCA1.2* may detect changes in cellular osmotic potential (Hou et al. 2014).

*ATX2* is a candidate for intermediate signalling. Within the ATX family, ATX1, ATX4, and ATX5—but not ATX3—are known to be important for ABA‐dependent drought response (Ding et al. 2011; Liu et al. 2018). *ATX2* has not been tested in dehydration response.

In this study, we evaluated the five candidates for roles in rapid high VPD response in the model plant Arabidopsis. Using T‐DNA insertion mutants, we examined leaf water potential and *AtNCED3* induction relative to WT during the first 20 min of high VPD exposure. The results suggest important roles for AT1G34300—which we named *HIGH VPD RESPONSE* (*HVR*)—and *MSL2*, while other candidates were not critical for high VPD‐induced upregulation of *AtNCED3*. These results provide new insights into the core components of this important rapid high VPD response pathway.

For each genotype (Table S1), we investigated plant water status by measuring the leaf water potential at key time points under high VPD (Supplemental Information: Materials and Methods). Both WT ecotypes (Col‐0 and Ws) exhibited a significant decline at 8 min (*p* < 0.05, when compared to 0 min), before returning to initial levels by 20 min (Figure 1A ). This is consistent with previous studies, where initial high transpiration decreases water potential, then stabilisation upon stomatal closure (Cardoso et al. 2018). Only one mutant, *osca1.2*, had a similar pattern to Col‐0 WT (Figure 1A ), suggesting that mutation to *OSCA1*.2 does not disrupt the physiological response to high VPD. All other genotypes showed a different response to their corresponding WT (Figure 1A ). These results suggest that *ATX2, HVR, ILK1*, and *MSL2* are important for the physiological response to high VPD.

**Figure 1:**
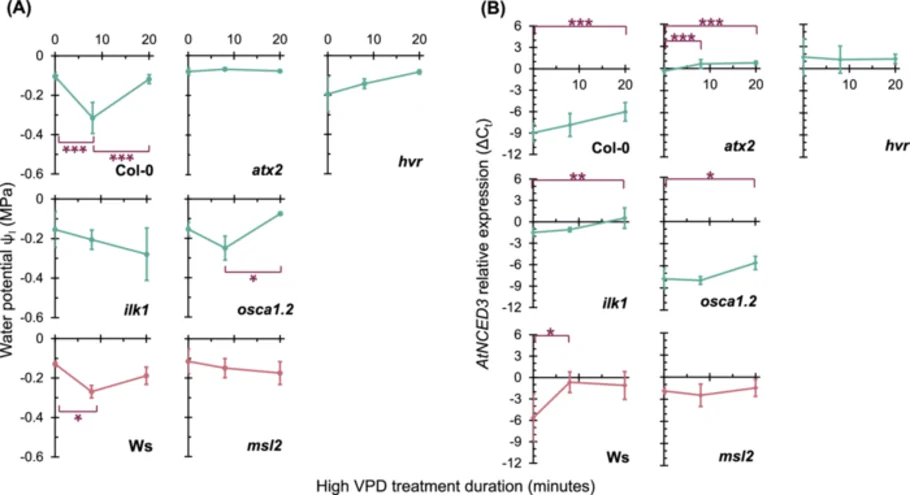
(A) Physiological response and (B) *AtNCED3* expression (ΔCt) in wild‐type (Col‐0, Ws) and mutant (*ilk1, osca1.2, hvr, atx2,* and *msl2*) *Arabidopsis thaliana* plants during high VPD response. Mean ± standard deviation are shown (*n* = 3‐4); asterisks (*) mark significant differences compared to 0 min within a genotype from Dunnett post hoc analysis: *p < 0.05, **p ≤ 0.01, ***p ≤ 0.001 (Table S3).

We next examined *AtNCED3* induction in high VPD (Figure 1B; Tables S2 and S3). In WT plants, *AtNCED3* expression increased significantly (Figure 1B), consistent with previous findings (Sussmilch et al. 2017). The induction pattern varied between the two ecotypes, with a significant induction peak at 8 min in Ws (*p* = 0.012), while Col‐0 exhibited more gradual induction that became significant after 20 min of high VPD (*p* < 0.001). Consistent with their WT counterpart, the *atx2, ilk1*, and *osca1.2* mutants showed significant upregulation of *AtNCED3* expression within 20 min (Figure 1B; Table S3), indicating these genes were not required for rapid *AtNCED3* induction in response to high VPD. However, baseline *AtNCED3* levels before treatment were elevated in *atx2* and *ilk1* relative to WT (Figure 1B), suggesting roles for *ATX2* and *ILK1* in maintaining low basal *AtNCED3* levels (Figure S1).

In contrast, *hvr* and *msl2* mutants did not exhibit *AtNCED3* induction (Figure 1B; Table S3), indicating that both genes are critical for rapid high VPD‐induced *AtNCED3* regulation. Additionally, both null mutants had higher baseline *AtNCED3* levels compared to WT, suggesting *HVR* and *MSL2* likely have roles in maintaining low *AtNCED3* levels under non‐stress conditions, but are deactivated in dehydration stress, allowing rapid *AtNCED3* upregulation (Figure S1). These findings are consistent with roles found in long‐term dehydration. In *msl2*, *AtNCED3* expression is consistently high in normal conditions, as plastid‐derived stress signals are continuously triggered (Wilson et al. 2014). Similarly, the same *hvr* mutant has a lower foliar water loss rate compared to WT (Kalladan et al. 2017). This suggests ABA biosynthesis and stomatal closure were triggered to maintain a high, constant water status in both normal and dehydrated conditions.

In conclusion, all tested mutants except *osca1.2* showed altered leaf water potential, indicating altered stomatal response compared to WT, suggesting *ATX2, HVR, ILK1*, and *MSL2* have roles in physiological response to high VPD. However, only *hvr* and *msl2* showed impairment to rapid *AtNCED3* induction, indicating *HVR* and *MSL2* are also critical for *AtNCED3* upregulation within 20 min of high VPD. The high basal *AtNCED3* expression in *atx2* and *ilk1* null mutants suggests they might function in preventing premature activation of *AtNCED3* under non‐stress conditions. In contrast, *OSCA1.2* is not critical for regulating these responses during high VPD stress. By identifying and validating the candidates, these findings illuminate key components of the rapid high VPD stress response pathway (Figure S1).

## Supporting information


Supporting File

## Data Availability

The data that support the findings of this study are available in the supporting material of this article.
