# Supplementary material for: Mutant Characterisation Reveals Two Candidate Sensors for the Rapid High Vapour Pressure Deficit (VPD) Response Pathway
Source: Plant Cell Environ. 2026 Aug 31;49(12):9732–4. doi: 10.1111/pce.70857 (PMC13725248; doi:10.1111/pce.70857)
Supplement: Supplementary file 1 [file PCE-49-9732-s001.docx]

**Mutant characterisation reveals two candidate sensors for the rapid high vapour pressure deficit (VPD) genetic response pathway**

Hanh M. Vo, Timothy J. Brodribb, Frances C. Sussmilch*

School of Natural Sciences, University of Tasmania, Private Bag 55, Hobart, TAS, 7001, Australia

Author email addresses: [hanhminh.vo@utas.edu.au](mailto:hanhminh.vo@utas.edu.au), [timothy.brodribb@utas.edu.au](mailto:timothy.brodribb@utas.edu.au), [frances.sussmilch@utas.edu.au*](mailto:frances.sussmilch@utas.edu.au*)

*Corresponding author

**ORCIDs:**

Hanh M. Vo: 0000-0002-3858-5775

Timothy J. Brodribb: 0000-0002-4964-6107

Frances Sussmilch: 0000-0002-8659-1125

**Funding:**

This research was funded by an Australian Research Council Discovery Early Career Researcher Award (DE200101133) from the Australian Government.

**Author contributions**

H.M.V, T.B, and F.C.S designed the research. H.M.V carried out the experiments, analysed the data and wrote the manuscript. T.B and F.C.S revised the manuscript.

**Data availability**

All data supporting the findings of this study are available within the manuscript and supplementary data.

**Conflict of Interest statement**

The authors declare no conflicts of interest.

**Keywords**

Abscisic acid (ABA) biosynthesis, *HIGH VPD RESPONSE* (*HVR*), *MECHANOSENSITIVE CHANNEL OF SMALL CONDUCTANCE-LIKE2* (*MSL2*), nine-*cis*-epoxycarotenoid dioxygenase (NCED), vapour pressure deficit (VPD).

**Abbreviations**

ATX2: ARABIDOPSIS TRITHORAX-LIKE2

HVR: HIGH VPD RESPONSE

ILK1: INTEGRIN-LINKED KINASE1

MSL2: MECHANOSENSITIVE CHANNEL OF SMALL CONDUCTANCE-LIKE2

NCED: NINE-*CIS-*EPOXYCAROTENOID DIOXYGENASE

OSCA1.2: REDUCED HYPEROSMOLALITY-INDUCED Ca^2+^ INCREASE 1.2

RLK: RECEPTOR-LIKE KINASE

VPD: vapour pressure deficit

WT: wild type

**Supplemental information:**

**Material and methods**

Plant growth and high VPD treatment

*Arabidopsis thaliana* wild-type – Colombia (Col-0) and Wassilewskija (Ws) –<https://www.arabidopsis.org/servlets/TairObject?type=species_variant&id=382> and mutant seeds were obtained from the Arabidopsis Biological Resource Centre (ABRC, The Ohio State University, Ohio, USA, <https://abrc.osu.edu/>), and grown on half-strength (0.5×) Murashige and Skoog (MS) basal media (Sigma-Aldrich, Missouri, USA) with additional sugar and selection additives for screening of mutant plants (**Table** **S1**). Mutants were confirmed with DNA genotyping (primer details in **Table S1**). The 14-day-old plants were transferred to standard potting mix soil, mixed with standard slow releasing fertilizer (Scott’s Osmocote®, Love The Garden, Bella Vista, New South Wales, Australia) according to the manufacturer’s instructions in 9 cm diameter pots and grown under short day standard glasshouse conditions (8 hours light/16 hours dark) at 25/20°C day/night. Plants were watered by filling trays underneath the pots to a depth of approximately 1 cm of water daily.

Six-week-old plants were covered overnight using transparent plastic bags to ensure plants were at full turgor and acclimated to a VPD of 0.46 ± 0.01 kPa. For high VPD treatment, the plastic bag was removed, and plants were exposed to 1.43 ± 0.17 kPa, maintained with a dehumidifier (CF08E, De’Longhi, Treviso, Italy). Leaves were collected at 0, 8 and 20 minutes of high VPD-exposure for (i) water potential quantification using a Scholander pressure chamber (Tyree & Hammel, 1972), and (ii) RNA extraction and analysis of *AtNCED3* gene expression.

Gene expression and statistical analysis

RNA was extracted with the Isolate II RNA Plant Kit (Meridian Bioscience, Ohio, USA) as previously described (Vo et al., 2025). RNA quantification, reverse transcription, and quantitative reverse transcription PCR (qRT-PCR) were conducted as previously described (McAdam et al., 2016; Sussmilch et al., 2017). Two housekeeping genes were tested (*AtMON1* and *AtUBQ10*). Based on the results from BestKeep© (Pfaffl et al., 2004), *AtMON1* was chosen as the most stable housekeeping gene for normalisation of *AtNCED3* expression. All qRT-PCR primer details are provided in **Table S2.** At least three replicates per timepoint for each genotype were used. Dunnett post-hoc analysis (Dunnett, 1955, 1964) was carried out in R v.4.2.3 and RStudio v.2024.12.0.467 (Posit team, 2024; R Core Team, 2023), using Tidyverse package (Wickham et al., 2019). The *AtNCED3* expression data and *P-*values are summarised in **Table S3**.

 **Fig. S1:** A schematic of the high VPD genetic response pathway in Arabidopsis, outlining the proposed roles of HVR, ILK1, MSL2 and ATX2 under normal and high VPD conditions, based on the results of this study. In non-stress conditions, the activity of transcription factor(s) are likely repressed indirectly (broken line) by MSL2, HVR, and ILK1, and directly/indirectly by ATX2 (solid line), thus maintaining low basal *NCED3* expression (**Fig. 1B**). Based on previous research, transcription factors implicated in the regulation of *NCED3* expression include: ARABIDOPSIS NAC DOMAIN CONTAINING PROTEIN2 (ANAC2/ATAF1; Jensen et al., 2013), NGATHA1 (NGA1; Sato et al., 2018), and WRKY57 (Jiang et al., 2012). Under high VPD, high transpiration rate decreases leaf turgor, which de-activates the repressive action of HVR and ILK1 for release of *NCED3* induction and physiological response (**Fig. 1**). The repression of *NCED3* by ATX2 is also released (**Fig. 1**), possibly through histone modification (Saleh et al., 2008). Excessive water loss also alters chloroplast osmotic potential, which likely triggers MSL2 for chloroplast osmotic regulation (Wilson et al., 2014), and also releases repression of *NCED3* expression by MSL2 (**Fig. 1**). This in turn catalyses the rate-limiting step in ABA biosynthesis converting 9’-*cis-*violaxanthin or 9’-*cis-*neoxanthin to xanthoxin (Qin & Zeevaart, 1999), triggering stomatal closure and limiting further water loss. Our experiments indicated that OSCA1.2 does not have a critical role in in controlling water potential or *AtNCED3* expression under high VPD (**Fig. 1**), and thus is not included in this proposed pathway

**Supplementary Figure and Tables:**

**Table S1:** Candidate gene information, mutant selection media and primer details. Primer details for DNA genotyping. Any primers that are from this study were designed with T-DNA Primer design Tool (Salk Institute Genome Analysis Laboratory (SIGnAL), 2003), or Primer3 (Kõressaar et al., 2018; Kõressaar & Remm, 2007; Untergasser et al., 2012), unless stated otherwise.

| **Candidate gene name** | **Background ecotype** | **Gene ID** | **ABRC mutant accession number** | **Selection media** | **Genotype and expected length** | **Primer sequences (5' to 3')** | **Tm (°C)** | **Primer source** |
| --- | --- | --- | --- | --- | --- | --- | --- | --- |
| *ARABIDOPSIS TRITHORAX-LIKE PROTEIN2* (*ATX2*) | Col-0 | AT1G05830 | *atx2-1*: SALK_074806C | - | Wild-type  1187 | TTCATGGTTTTGGGATCTTTG | 55 | This study, designed with T-DNA Primer design Tool |
|  |  |  |  |  |  | TTCCTCACCATGAAATAACGC | 55 |  |
|  |  |  |  | Kanamycin | Mutant  ~760 | TTCATGGTTTTGGGATCTTTG | 55 |  |
|  |  |  |  |  |  | ATTTTGCCGATTTCGGAAC | 55 | LBb1.3 pROK2 (Salk Institute Genome Analysis Laboratory (SIGnAL), 2003) |
| *HIGH VPD RESPONSE* (*HVR*) | Col-0 | AT1G34300 | SALK_132270C | - | Wild-type  820 | TCTCTACCATACCTCTCGGC | 55 | This study, designed with Primer3 |
|  |  |  |  |  |  | TCAAAGTTACGAGACGGACAC | 55 |  |
|  |  |  |  | Kanamycin | Mutant  ~750 | TCTCTACCATACCTCTCGGC | 55 |  |
|  |  |  |  |  |  | ATTTTGCCGATTTCGGAAC | 55 | LBb1.3 pROK2 (Salk Institute Genome Analysis Laboratory (SIGnAL), 2003) |
| *INTEGRIN-LINKED KINASE1* (*ILK1*) | Col-0 | AT2G43850 | *ilk1-2*: CS864891/ WiscDsLoxHs009_11D | - | Wild-type  1801 | TGAGAGATATGGACCAAACAACT | 55 | This study, designed with T-DNA Primer design Tool |
|  |  |  |  |  |  | CCAGAAAGACAAAGCAGAATC | 55 | (Brauer et al., 2016) |
|  |  |  |  | BASTA (glufosinate-ammodium) | Mutant  ~1119 | TGAGAGATATGGACCAAACAACT | 55 | This study, designed with T-DNA Primer design Tool |
|  |  |  |  |  |  | AATAGCCTTTACTTGAGTTGGCGTAAAAG | 55 | LT6pDsLox (Woody et al., 2007) |
| *MECHANO-SENSITIVE CHANNEL OF SMALL CONDUCTANCE-LIKE2* (*MSL2*) | Ws | AT5G10490 | *msl2-3*: CS69611/ GK-195D11 | - | Wild-type  3560 | GTTGGGACAACATGGTTTCAG | 55 | AtMSL2-3_GK-195D11RP, AtMS2-WISB, JL-202 respectively  (Haswell & Meyerowitz, 2006) |
|  |  |  |  |  |  | TTACATCATGACTAGAGACAGGTAAGCTA | 55 |  |
|  |  |  |  | Sulfadiazine | Mutant  ~598 | GTTGGGACAACATGGTTTCAG | 55 |  |
|  |  |  |  |  |  | CATTTTATAATAACGCTGCGGACATCTAC | 55 |  |
| *HYPER-OSMOLALITY-INDUCED Ca2+ INCREASE1.2* (*OSCA1.2*) | Col-0 | AT4G22120 | *osca1.2*: SALK_200646C | - | Wild-type  1155 | AGCTCAGGGCCACTTTTTGT | 55 | This study, designed with T-DNA Primer design Tool |
|  |  |  |  |  |  | GGGTCTGTTGAGTCTGAGCAC | 55 |  |
|  |  |  |  | Kanamycin | Mutant  ~800 | GGGTCTGTTGAGTCTGAGCAC | 55 |  |
|  |  |  |  |  |  | ATTTTGCCGATTTCGGAAC | 55 | LBb1.3 pROK2 (Salk Institute Genome Analysis Laboratory (SIGnAL), 2003) |

**Table S2**: Primer details for qRT-PCR experiments presented in **Fig. 1.**

| **Gene name** | **Accession number** | **Primer sequences (5' to 3')** | **Tm (°C)** | **Primer source** |
| --- | --- | --- | --- | --- |
| *AtNCED3* | AT3G14440 | CGGTGGTTTACGACAAGAACAA | 60.0 | (Barrero et al., 2006) |
|  |  | CAGAAGCAATCTGGAGCATCAA |  |  |
| *MONENSIN SENSITIVITY1* (*AtMON1*) | AT2G28390 | AACTCTATGCAGCATTTGATCCACT | 60.0 | (Czechowski et al., 2005) |
|  |  | TGATTGCATATCTTTATCGCCATC |  |  |
| *POLYUBIQUITIN10* (*AtUBQ10*) | AT4G05320 | GGCCTTGTATAATCCCTGATGAATAAG | 60.0 | (Czechowski et al., 2005) |
|  |  | AAAGAGATAACAGGAACGGAAACATAGT |  |  |

**Table S3:** *AtNCED3* expression data for **Fig. 1**. *AtNCED3* gene expression values were normalised against the house-keeping gene *AtMON1* (ΔC_T_ ± standard deviation) and timepoint 0 for each genotype (relative *AtNCED3* expression, ΔΔC_T_ ± standard deviation). One way ANOVA was performed; when ANOVA results showed significant differences between timepoints, Dunnett's multiple comparison test (Dunnett, 1964) was performed for 8 and 20 minutes relative to 0 minutes for each genotype.

| **Genotype (background)** | **High VPD treatment duration (min)** | ***AtNCED3* expression normalised against house-keeper *AtMON1* (ΔC_T_)** | **Relative *AtNCED3* expression normalised against 0 minutes for each genotype (ΔΔC_T_)** | **ANOVA *p*-value** | **Dunnet test *p*-value** | **Significance level** |
| --- | --- | --- | --- | --- | --- | --- |
| WT (Col-0) | 0 | -8.974 ± 1.318 | 0.000 ± 0.858 |  | - |  |
|  | 8 | -7.870 ± 1.609 | 1.234 ± 1.664 | < 0.001 | 0.631 |  |
|  | 20 | -6.025 ± 1.297 | 3.079 ± 0.953 |  | < 0.001 | ******* |
| *atx2* (Col-0) | 0 | -0.355 ± 0.454 | 0.000 ± 0 |  | - |  |
|  | 8 | 0.618 ± 0.596 | 0.973 ± 0.146 | < 0.001 | < 0.001 | ******* |
|  | 20 | 0.767 ± 0.232 | 1.122 ± 0.227 |  | < 0.001 | ******* |
| *hvr* (Col-0) | 0 | 1.575 ± 2.478 | 0.000 ± 0 |  | - |  |
|  | 8 | 1.232 ± 1.840 | -0.343 ± 0.872 | 0.638 | - |  |
|  | 20 | 1.318 ± 0.638 | -0.257 ± 2.819 |  | - |  |
| *ilk1* (Col-0) | 0 | -1.485 ± 0.454 | 0.000 ± 0 |  | - |  |
|  | 8 | -1.093 ± 0.596 | 0.392 ± 0.342 | < 0.001 | 0.678 |  |
|  | 20 | 0.532 ± 1.444 | 2.017 ± 0.894 |  | 0.010 | ****** |
| *osca1.2* (Col-0) | 0 | -7.762 ± 1.365 | 0.000 ± 0.788 |  | - |  |
|  | 8 | -7.982 ± 0.535 | -0.220 ± 0.309 | < 0.05 | 0.946 |  |
|  | 20 | -5.603 ± 0.905 | 2.159 ± 0.453 |  | 0.0414 | ***** |
| WT (WS) | 0 | -5.635 ± 3.317 | 0.000 ± 0 |  | - |  |
|  | 8 | -0.657 ± 1.457 | 4.978 ± 1.951 | < 0.05 | 0.012 | ***** |
|  | 20 | -1.108 ± 1.951 | 4.527 ± 4.068 |  | 0.126 |  |
| *msl2* (WS) | 0 | -1.868 ± 0.410 | 0.000 ± 0.410 |  | - |  |
|  | 8 | -2.460 ± 1.520 | -0.592 ± 1.520 | 0.582 | - |  |
|  | 20 | -1.463 ± 1.154 | 0.405 ±1.154 |  | - |  |

**Supplementary references**

Barrero, J. M., Rodríguez, P. L., Quesada, V., Piqueras, P., Ponce, M. R., & Micol, J. L. (2006). Both abscisic acid (ABA)-dependent and ABA-independent pathways govern the induction of *NCED3*, *AAO3* and *ABA1* in response to salt stress. *Plant, Cell and Environment*, *29*(10), 2000–2008. https://doi.org/10.1111/j.1365-3040.2006.01576.x

Brauer, E. K., Ahsan, N., Dale, R., Kato, N., Coluccio, A. E., Piñeros, M. A., Kochian, L. V, Thelen, J. J., & Popescu, S. C. (2016). The Raf-like kinase ILK1 and the high affinity K^+^ transporter HAK5 are required for innate immunity and abiotic stress response. *Plant Physiology*, *171*(2), 1470–1484. https://doi.org/10.1104/pp.16.00035

Czechowski, T., Stitt, M., Altmann, T., & Udvardi, M. K. (2005). Genome-wide identification and testing of superior reference genes for transcript normalization in Arabidopsis. *Plant Physiology*, *139*(1), 5–17. https://doi.org/10.1104/pp.105.063743.1

Dunnett, C. W. (1955). A multiple comparison procedure for comparing several treatments with a control. *Journal of the American Statistical Association*, *50*(272), 1096–1121. https://doi.org/10.1080/01621459.1955.10501294

Dunnett, C. W. (1964). New tables for multiple comparisons with a control. *Biometrics*, *20*(3), 482–491. https://doi.org/https://doi.org/10.2307/2528490

Haswell, E. S., & Meyerowitz, E. M. (2006). MscS-like proteins control plastid size and shape in *Arabidopsis thaliana*. *Current Biology*, *16*(1), 1–11. https://doi.org/10.1016/j.cub.2005.11.044

Jensen, M. K., Lindemose, S., Masi, F. de, Reimer, J. J., Nielsen, M., Perera, V., Workman, C. T., Turck, F., Grant, M. R., Mundy, J., Petersen, M., & Skriver, K. (2013). ATAF1 transcription factor directly regulates abscisic acid biosynthetic gene *NCED3* in *Arabidopsis thaliana*. *FEBS Open Bio*, *3*, 321–327. https://doi.org/10.1016/j.fob.2013.07.006

Jiang, Y., Liang, G., & Yu, D. (2012). Activated expression of *WRKY57* confers drought tolerance in Arabidopsis. *Molecular Plant*, *5*(6), 1375–1388. https://doi.org/10.1093/mp/sss080

Kõressaar, T., Lepamets, M., Kaplinski, L., Raime, K., Andreson, R., & Remm, M. (2018). Primer3_masker: integrating masking of template sequence with primer design software. *Bioinformatics (Oxford, England)*, *34*(11), 1937–1938. https://doi.org/10.1093/bioinformatics/bty036

Kõressaar, T., & Remm, M. (2007). Enhancements and modifications of primer design program Primer3. *Bioinformatics (Oxford, England)*, *23*(10), 1289–1291. https://doi.org/10.1093/bioinformatics/btm091

McAdam, S. A. M., Sussmilch, F. C., & Brodribb, T. J. (2016). Stomatal responses to vapour pressure deficit are regulated by high speed gene expression in angiosperms. *Plant Cell and Environment*, *39*(3), 485–491. https://doi.org/10.1111/pce.12633

Pfaffl, M. W., Tichopad, A., Prgomet, C., & Neuvians, T. P. (2004). Determination of most stable housekeeping genes, differentially regulated target genes and sample integrity : BestKeeper© - Excel spreadsheet tool using a Repeated Pair-wise Correlation and Regression Analysis. *Biotechnology Letters*, 509–515.

Posit team. (2024). *RStudio: Integrated development for R*. Posit Software, PBC, Boston, MA.

Qin, X., & Zeevaart, J. A. D. (1999). The 9-*cis*-epoxycarotenoid cleavage reaction is the key regulatory step of abscisic acid biosynthesis in water-stressed bean. *Proceedings of the National Academy of Sciences*, *96*(26), 15354–15361. https://doi.org/10.1073/pnas.96.26.15354

R Core Team. (2023). *R: A language and environment for statistical computing*.

Saleh, A., Alvarez-Venegas, R., Yilmaz, M., Le, O., Hou, G., Sadder, M., Al-Abdallat, A., Xia, Y., Lu, G., & Ladunga, I. (2008). The highly similar Arabidopsis homologs of trithorax *ATX1* and *ATX2* encode proteins with divergent biochemical functions. *The Plant Cell*, *20*(3), 568–579.

Salk Institute Genome Analysis Laboratory (SIGnAL). (2003). *T-DNA Primer Design*. http://signal.salk.edu/tdnaprimers.2.html

Sato, H., Takasaki, H., Takahashi, F., Suzuki, T., Iuchi, S., & Mitsuda, N. (2018). *Arabidopsis thaliana* NGATHA1 transcription factor induces ABA biosynthesis by activating *NCED3* gene during dehydration stress. *Proceedings of the National Academy of Sciences*, *115*(47), E11178–E11187. https://doi.org/10.1073/pnas.1811491115

Sussmilch, F. C., Brodribb, T. J., & McAdam, S. A. M. (2017). Up-regulation of *NCED3* and ABA biosynthesis occur within minutes of a decrease in leaf turgor but *AHK1* is not required. *Journal of Experimental Botany*, *68*(11), 2913–2918. https://doi.org/10.1093/jxb/erx124

Tyree, M. T., & Hammel, H. T. (1972). The measurement of the turgor pressure and the water relations of plants by the pressure-bomb technique. *Journal of Experimental Botany*, *23*(1), 267–282. https://doi.org/10.1093/jxb/23.1.267

Untergasser, A., Cutcutache, I., Koressaar, T., Ye, J., Faircloth, B. C., Remm, M., & Rozen, S. G. (2012). Primer3-new capabilities and interfaces. *Nucleic Acids Research*, *40*(15), e115. https://doi.org/10.1093/nar/gks596

Vo, H. M., Charleston, M. A., Brodribb, T. J., & Sussmilch, F. C. (2025). Dehydration rapidly induces expression of *NCED* genes from a single subclade in diverse eudicots. *Planta*, *261*(2), 46. https://doi.org/10.1007/s00425-025-04626-z

Wickham, H., Averick, M., Bryan, J., Chang, W., McGowan, François, Grolemund, Hayes, Henry, L., Hester, J., Kuhn, M., Pedersen, T. L., Miller, E., Bache, S. M., Müller, K., Ooms, J., Robinson, D., Seidel, D. P., Spinu, V., … Yutani, H. (2019). Welcome to the Tidyverse. *Journal of Open Source Software*, *4*(43), 1686. https://doi.org/10.21105/joss.01686

Wilson, M. E., Basu, M. R., Bhaskara, G. B., Verslues, P. E., Haswell, E. S., Louis, S., & Missouri, M. E. W. (2014). Plastid osmotic stress activates cellular stress responses in Arabidopsis. *Plant Physiology*, *165*(1), 119–128. https://doi.org/10.1104/pp.114.236620

Woody, S. T., Austin-Phillips, S., Amasino, R. M., & Krysan, P. J. (2007). The WiscDsLox T-DNA collection: an Arabidopsis community resource generated by using an improved high-throughput T-DNA sequencing pipeline. *Journal of Plant Research*, *120*(1), 157–165. https://doi.org/10.1007/s10265-006-0048-x
